# Dynamics in gut microbiota diversity, composition, and assembly reveal the adaptability of invasive snail *Pomacea canaliculata* during hibernation in rice fields

**DOI:** 10.3389/fmicb.2025.1616681

**Published:** 2025-07-16

**Authors:** Fucheng Yao, Chuang Li, Yingtong Chen, Jiaen Zhang, Zhaoji Shi, Zhong Qin

**Affiliations:** ^1^Department of Ecology, College of Natural Resources and Environment, South China Agricultural University, Guangzhou, China; ^2^Guangdong Laboratory for Lingnan Modern Agriculture, South China Agricultural University, Guangzhou, China; ^3^Guangdong Engineering Technology Research Centre of Modern Eco-agriculture and Circular Agriculture, South China Agricultural University, Guangzhou, China; ^4^Key Laboratory of Agro-Environment in the Tropics, Ministry of Agriculture and Rural Affairs, South China Agricultural University, Guangzhou, China

**Keywords:** invasive alien species, *Pomacea canaliculata*, gut microbiota, hibernation, community assembly

## Abstract

The gut microbiota plays a crucial role in host immunity and metabolism and may facilitate the adaptation of invasive species to new environments. During hibernation, gut microbial communities undergo compositional shifts to help hosts cope with low temperatures and food scarcity. However, the dynamics of gut microbiota during hibernation in invasive animals remain poorly understood. Here, we conducted an *in situ* hibernation experiment on the invasive freshwater snail *Pomacea canaliculata* to investigate changes in its gut microbiota over the course of hibernation. Gut samples were collected at pre-hibernation (day 0) and on the 15th, 30th, 60th, 90th, and 120th days of hibernation, followed by 16S rRNA gene sequencing. Results showed that the survival rate of snails reached 85.7% after 120 days. The Shannon diversity index of gut microbiota increased with the duration of hibernation. Although species richness remained relatively stable, increased evenness led to higher alpha diversity. After 60 days of hibernation, the structure of gut microbial community changed. The dominant phylum shifted from *Firmicutes* to *Bacteroidota* (formerly *Bacteroidetes*) as hibernation progressed. Short chain fatty acids (SCFAs) producing genera such as *Acetobacteroides*, *Bacteroides*, *Macellibacteroides*, and *Cetobacterium* increased in abundance during hibernation, likely providing an energy source for both the gut and host. Gut microbiota changes appeared to be driven largely by stochastic assembly processes. Additionally, anaerobic bacteria and potential pathogens increased in abundance during hibernation. These adaptive shifts in gut microbiota may help maintain host metabolic and immune functions during hibernation and potentially contribute to the invasiveness of *P. canaliculata*.

## 1 Introduction

*Pomacea canaliculata* (Ampullariidae), commonly known as apple snails and native to the Río de la Plata basin in South America, has become an invasive agricultural pest across Africa, Asia, and southern regions of North America (Seuffert and Martín, 2021; Constantine et al., 2023; Yao et al., 2023). This voracious snail feeds on rice and other aquatic crops, posing a major threat to agriculture and food security (Hayes et al., 2008; Horgan et al., 2021). Furthermore, *P. canaliculata* causes severe biodiversity loss and disrupts the functions of wetland ecosystems (Fang et al., 2010; O'Neil et al., 2023). Additionally, the snails harbor numerous *Angiostrongylus cantonensis* (rat lungworm) and other pathogens, which severely impact human health (Song et al., 2016). Therefore, elucidating the invasion mechanisms of *P. canaliculata* and developing effective control strategies are urgently needed for sustainable agricultural development and ecosystem protection.

*P. canaliculata* exhibits high environmental plasticity, enabling it to withstand adverse conditions such as low temperatures, desiccation, and food deprivation (Lach et al., 2000; Yusa et al., 2006; Wada and Matsukura, 2007; Wada and Matsukura, 2011). Adverse environmental conditions such as low water levels, extreme temperatures, or food scarcity can cause *P. canaliculata* to bury itself in the soil and enter a state of dormancy (Lach et al., 2000; Wada and Yoshida, 2000; Ito, 2002). After the late rice harvest in winter, as paddy fields dry up and temperatures drop, *P. canaliculata* burrows into the surface soil to overwinter (Hayes et al., 2015). The snails remain dormant in the soil until irrigation resumes the next year, after which they crawl out of the soil and resume their activities. This overwintering phenomenon is commonly referred to as ‘hibernation’ (Hayes et al., 2015). Short-term hibernation experiments on *P. canaliculata* revealed elevated levels of tissue antioxidants, such as uric acid and glutathione (GSH), indicating an enhanced endogenous antioxidant defense mechanism for protection during hibernation (Giraud-Billoud et al., 2018; Rodriguez et al., 2023). Furthermore, *P. canaliculata* increases its own antioxidant enzyme activity to cope with oxidative stress during the in-situ hibernation period in rice fields. When exposed to cold waves, they regulate cold-tolerance related substances (e.g., glycerol, bound water, etc.) in their bodies to adapt. Moreover, the survival rate of snails exhibits a female advantage (Yao et al., 2024). Successful overwintering in new habitats is a key factor for the invasive spread of *P. canaliculata* into the middle temperate zone. Therefore, it is necessary to further explore the mechanisms related to its hibernation.

Gut microbiota technologies have been applied to elucidate the invasion mechanisms of *P. canaliculata*. Liu et al. (2018) indicated that the gut microbiome of *P. canaliculata* plays key roles in stress resilience and food digestion, as revealed by metagenomic analysis. Factors like age and sex significantly influence the gut microbiota composition of this snail (Chen et al., 2021). Zhou Z. et al. (2022) observed that *P. canaliculata* has greater richness of unique microbial taxa when compared with native Chinese snails (*Cipangopaludina chinensis*). Similarly, Shi et al. (2024) discovered that more deterministic assembly processes constrain the diversity of gut microbiota in *P. canaliculata* and the native snail (Viviparidae). The gut microbiota of *P. canaliculata* exhibits adaptive responses to seasonal and temperature fluctuations (Li et al., 2022a; Li et al., 2022b). Additionally, some studies reveal the tolerance of *P. canaliculata* to pollutants by examining changes in gut microbiota of snails (Bao et al., 2024; Bi et al., 2024).

The gut microbiota has been shown to influence host digestion, metabolism, immunity, and resistance to pathogens (Iebba et al., 2012; Sisa et al., 2017). During hibernation, certain gut microbes proliferate and enhance the synthesis of short chain fatty acids (SCFAs), such as acetate (Carey et al., 2013). These SCFAs serve as crucial energy sources for both intestinal epithelial cells and the host organism. The liver can utilize acetate transported from the gut to synthesize fatty acids and cholesterol (Macfarlane and Macfarlane, 2003). Hepatic and intestinal epithelial cells in mammals can also convert acetate into ketone bodies, thereby supplying energy to the brain, muscles, and heart during hibernation (Carey et al., 2003; Heldmaier et al., 2004). Additionally, the gut microbiota can help the host (e.g., sloths and arctic ground squirrel) resist microbial invasion during hibernation by producing organic acids, secreting antimicrobial compounds, and competing with pathogens (Stevenson et al., 2014; Dill-McFarland et al., 2016). However, the response and dynamic changes of the gut microbiota in *P. canaliculata* during hibernation remain unclear. Exploring the gut microbiota of *P. canaliculata* during hibernation could further reveal its invasion mechanisms.

Here, we investigated the gut bacteriome of *P. canaliculata* before and after 0, 15, 30, 60, 90, and 120 days of *in situ* overwintering in rice paddies. The specific objectives were: (1) examine changes in diversity and community composition of snail gut microbiota during hibernation; (2) identify dominant and key gut bacteria after hibernation; and (3) elucidate changes in assembly processes and phenotypes of the gut microbiome during post-hibernation.

## 2 Materials and methods

### 2.1 Experimental materials

*Pomacea canaliculata* snails were cultured in cement ponds located at the Ecological Teaching and Research Farm (23° 16′N, 113° 36′E) of South China Agricultural University (SCAU) in Guangzhou, China. The region experiences a humid subtropical monsoon climate. Yearly average temperature is 21.5°C, with January and July marking the coolest and warmest periods, respectively. Annual rainfall fluctuates between 1,612 and 1909 mm, predominantly occurring from April to September, which accounts for over 80% of the total precipitation. Prior to the experiment, these snails were transported to the laboratory for sex and size selection. After screening, female snails with a shell height of 30–35 mm were selected for *in situ* hibernation experiments.

### 2.2 Experimental design

**Figure 1:**
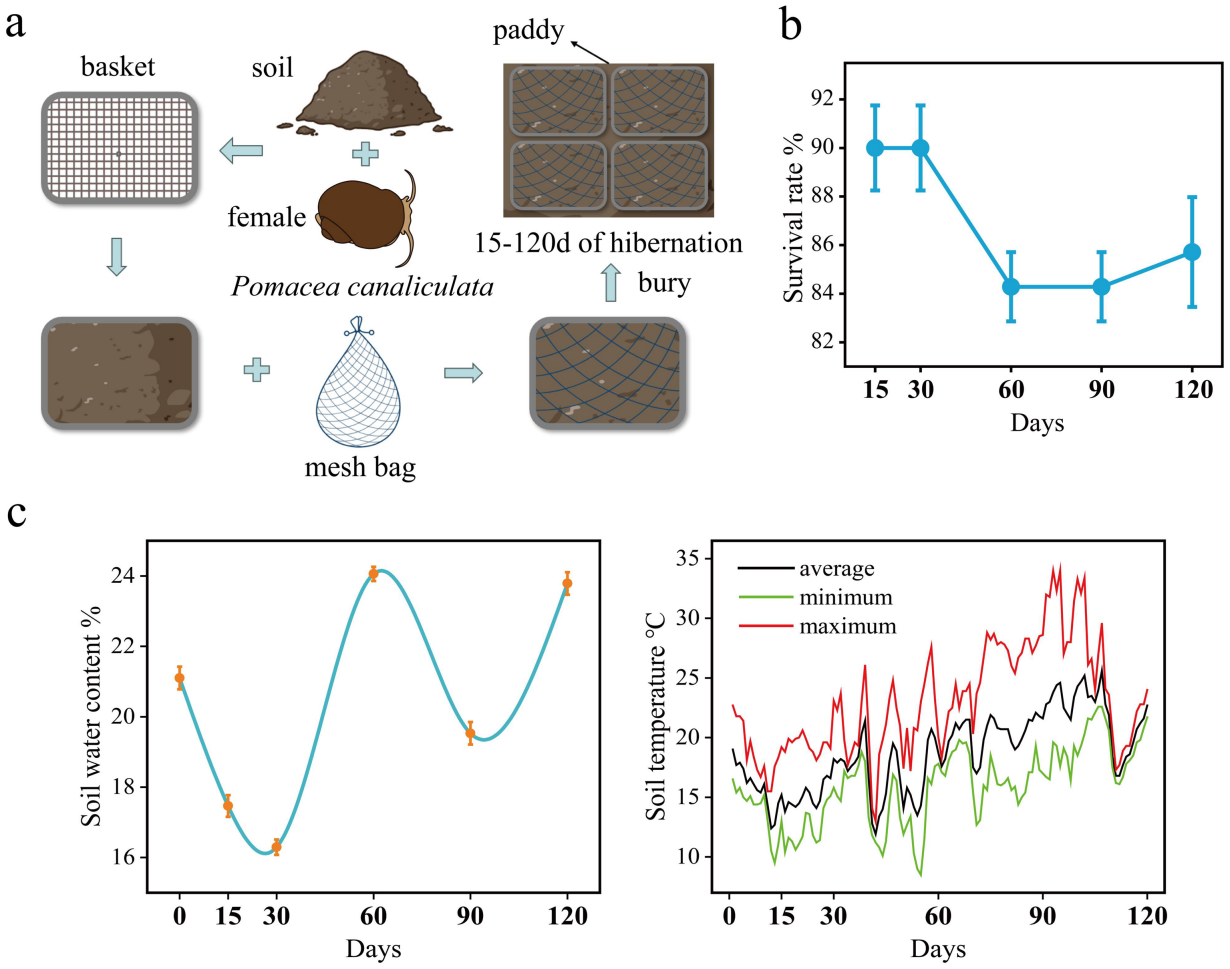
**(a)** Schematic diagram of *P. canaliculata* snails *in situ* hibernation experiment. **(b)** Survival rate of the snails during hibernation. **(c)** Soil temperature (right) and water content (left) during hibernation.

The in-situ hibernation experiment (Figure 1a) was conducted in the paddy field of the Ecological Teaching and Research Farm at SCAU from December 2022 to April 2023 (120 days in total). Specifically, a plastic mesh basket (31.5 cm × 23 cm × 10 cm) was filled with 5 cm of *in situ* soil which was taken from a depth of 5–10 cm, and then 14 female snails were evenly placed on the soil surface. Next, the basket was continued filling with soil from 0 to 5 cm depth until it was nearly full. Once the snails and soil were properly arranged, the basket was covered with a mesh bag to prevent snail escape. Finally, the entire basket was placed into pre-dug trenches approximately 10 cm deep, covering the surface with a small amount of soil to make it level with the ground.

A total of 25 baskets were set up for the in situ experiment. At 15th, 30th, 60th, 90th, and 120th days of the experiment, five randomly selected baskets were retrieved from the field and transported back to the laboratory for snail sampling. The buried female snails were collected by carefully removing the soil. The number of dead snails was recorded. From each basket, one snail was randomly selected and dissected on a sterile workbench. The dissected intestines (from the pylorus to the hindgut) were placed in 2 mL sterile cryovials (Bikeman Biotechnology Co., Ltd., Hunan, China). These cryovials containing the intestinal samples were immediately frozen in liquid nitrogen. Once the sampling process was completed, the cryovials were stored at −80°C for preservation.

### 2.3 DNA extraction and 16S rRNA sequencing

Microbial DNA was extracted from 30 intestinal samples (each sample represents the intestine of a single snail from a different basket) using the FastDNA® Kit (MP Biomedicals, CA, USA) according to the manufacturer’s protocol, respectively. The quality and quantity of DNA were evaluated by 1% agarose gel electrophoresis. A NanoDrop2000 spectrophotometer (Thermo Scientific, Wilmington, USA) was employed to determine DNA purity and concentration.

The V3-V4 region of the bacterial 16S rRNA genes was amplified using primers 338F (5′-ACTCCTACGGGAGGCAGCAG-3′) and 806R (5′-GGACTACHVGGGTWTCTAAT-3′). The amplified products were purified using the AxyPrepDNA kit (AXYGEN, USA) and quantified using the QuantiFluorTM-ST (Promega, USA). High-throughput sequencing of the PCR products was performed on an Illumina MiSeq PE300 platform at Majorbio BioPharm Technology Co., Ltd. (Shanghai, China).

### 2.4 Bioinformatic analysis

Raw data was subjected to bioinformatics analysis using QIIME 1.9.1 software. The raw fastq files were processed for demultiplexing and denoising using FLASH 1.2.11 and Trimmomatic, respectively. The sequence data were assigned to operational taxonomic units (OTUs) using USEARCH 7.1 software with a 97% identity threshold (Edgar et al., 2011). RDP Classifier 2.13 was utilized to assign taxonomy to each 16S rRNA gene sequence by comparing it against the Silva 138 (rRNA database) (Quast et al., 2012; Bokulich et al., 2018). The taxonomic identity of the unranked OTUs at the genus level was determined by querying them against the NCBI database using BLAST. Alpha-diversity indices (Chao1, Shannon, Simpson (not Gini-Simpsion), PD, and Pielou evenness) of the microbial community were calculated using QIIME. Phenotypic properties (Gram Negative, Gram Positive, Pathogenic, Mobile Element Containing, Oxygen Utilizing, Biofilm Forming, and Oxidative Stress Tolerant) were performed using Bugbase software (Zhang et al., 2019).

### 2.5 Soil temperature and water content measurement

Soil temperature was monitored in real-time during the in-situ experiment using a temperature intelligent monitoring device (developed by CIMC Intelligent Cold Chain Technology, Beijing, China), which uploaded temperature data every hour. The temperature probe was placed at a 5 cm depth in the soil of the experimental site. During each sampling event, soil samples from the 0–10 cm depth were collected and brought back to the laboratory. Soil water content was determined by drying samples at 105°C.

### 2.6 Survival rate assay

At each sampling time point (15th, 30th, 60th, 90th, and 120th days), five baskets were collected and transported to the laboratory. The female snails were delicately separated from the soil, and dead individuals were recorded. The survival of each snail was determined by assessing the presence of odor indicating decay or gently testing whether the operculum would retract upon light touch. Finally, the survival rate of the snails was calculated.

### 2.7 Statistical analysis

Beta diversity was analyzed using non-metric multidimensional scaling (NMDS). The vegan package was used for permutational multivariate analysis of variance (PERMANOVA), with Bray–Curtis dissimilarity as the distance measure. Intestinal microbial composition differences between hibernation periods were analyzed using Linear Discriminant Analysis (LDA) Effect Size (LEfSe), considering only those features with an absolute LDA score greater than 4 (*p* < 0.05) (Li et al., 2022b). The relationship between hibernation duration and specific phyla, genera, alpha diversity indices, and phenotypic properties was established using generalized additive models (GAMs). The ecological process of community assembly was estimated using the iCAMP R package’s phylogenetic bin-based null model (Ning et al., 2020). The normalized stochasticity ratio (NST) was used to quantify the relative importance of stochastic and deterministic processes in gut microbiota assembly, with a threshold of 50% set to determine the dominance of either deterministic or stochastic processes (Ning et al., 2019).

## 3 Results

### 3.1 Hibernation environment and survival rate

During the 120-day hibernation period, the average soil temperature was 18.66°C. The average soil temperatures for the periods of day 0–30, day 30–60, day 60–90, and day 90–120 were 15.5°C, 16.7°C, 20.3°C, and 22°C, respectively. The lowest soil temperature occurred on the 55th day, reaching 8.5°C (Figure 1c). The highest soil temperature was recorded on the 93rd and 95th days, reaching 34°C (Figure 1c). Soil moisture content was at its lowest on the 30th day, at 16.3%, and reached its peak on the 60th day, at 24.1% (Figure 1c). After 120 days of hibernation, the survival rate of female snails was 85.7% (Figure 1b).

### 3.2 Sequencing depth and alpha diversity indices

A total of 30 snail gut samples, collected from 6 periods (0, 15th, 30th, 60th, 90th, and 120th days), underwent 16S rRNA high-throughput sequencing analysis. The analysis yielded 2,443,935 valid sequences, identifying 2,821 OTUs at a 97% similarity threshold. The species accumulation curve of all samples (Supplementary Figure S1) demonstrated that the observed species richness approached saturation, indicating the reliability of sequencing data and its suitability for subsequent analysis.

**Figure 2:**
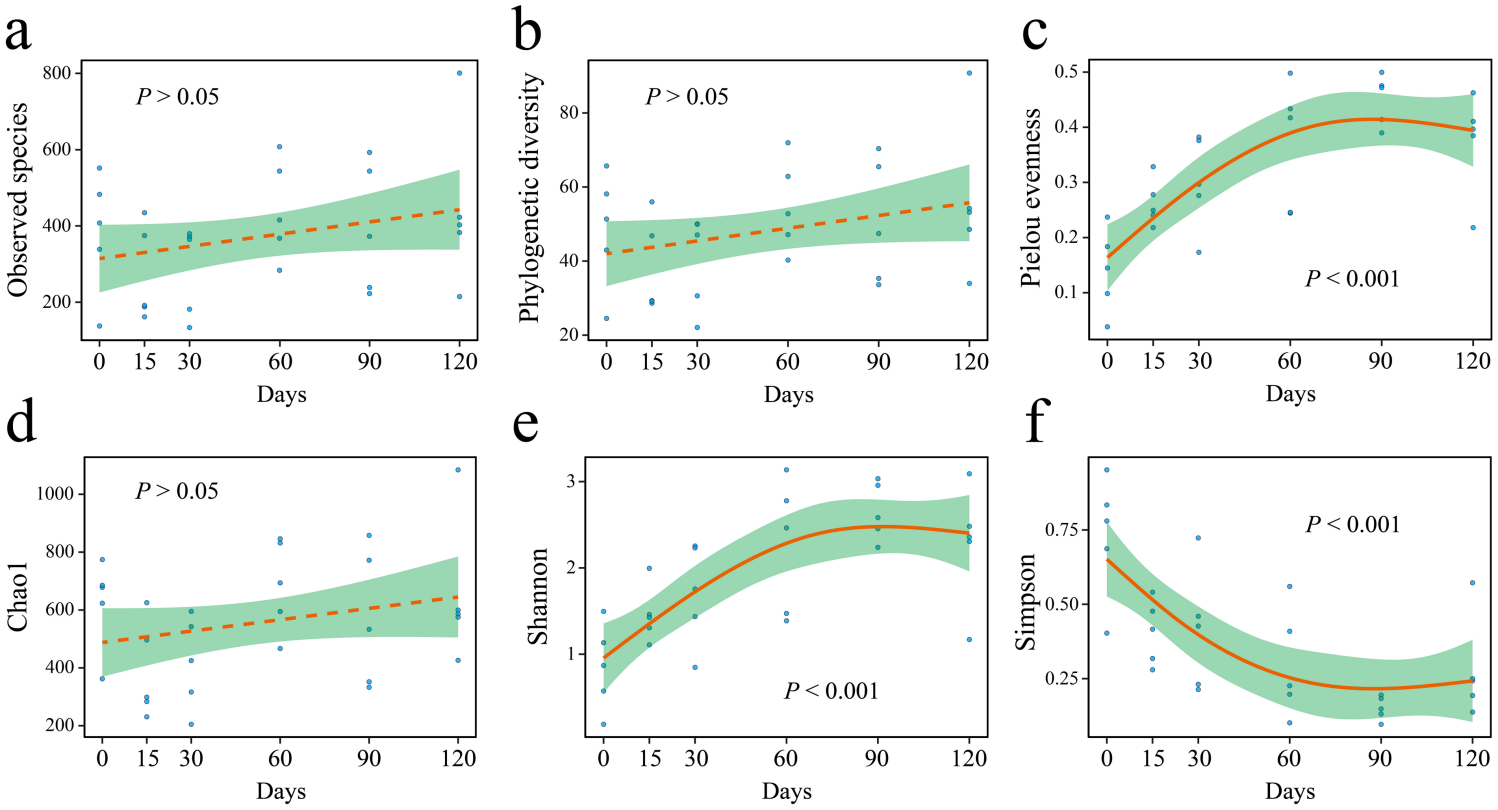
Alpha diversity indices **(a–f)** of gut microbiota in *P. canaliculata* snails during 0–120 days of hibernation.

The relationship between microbial diversity indices and hibernation duration was analyzed using the generalized additive model (Figure 2). Hibernation duration had no significant effect on the Sobs (observed species), phylogenetic diversity (PD), or Chao1 indices (Figures 2a,b,d). In contrast, the Pielou evenness, Shannon, and Simpson indices exhibited significant correlations with hibernation duration (Figures 2c,e,f). Specifically, both the Pielou evenness and Shannon indices increased over time (Figures 2c,e), indicating enhanced community evenness and diversity. Conversely, the Simpson index decreased with hibernation duration (Figure 2f).

### 3.3 Community composition and beta diversity analysis

**Figure 3:**
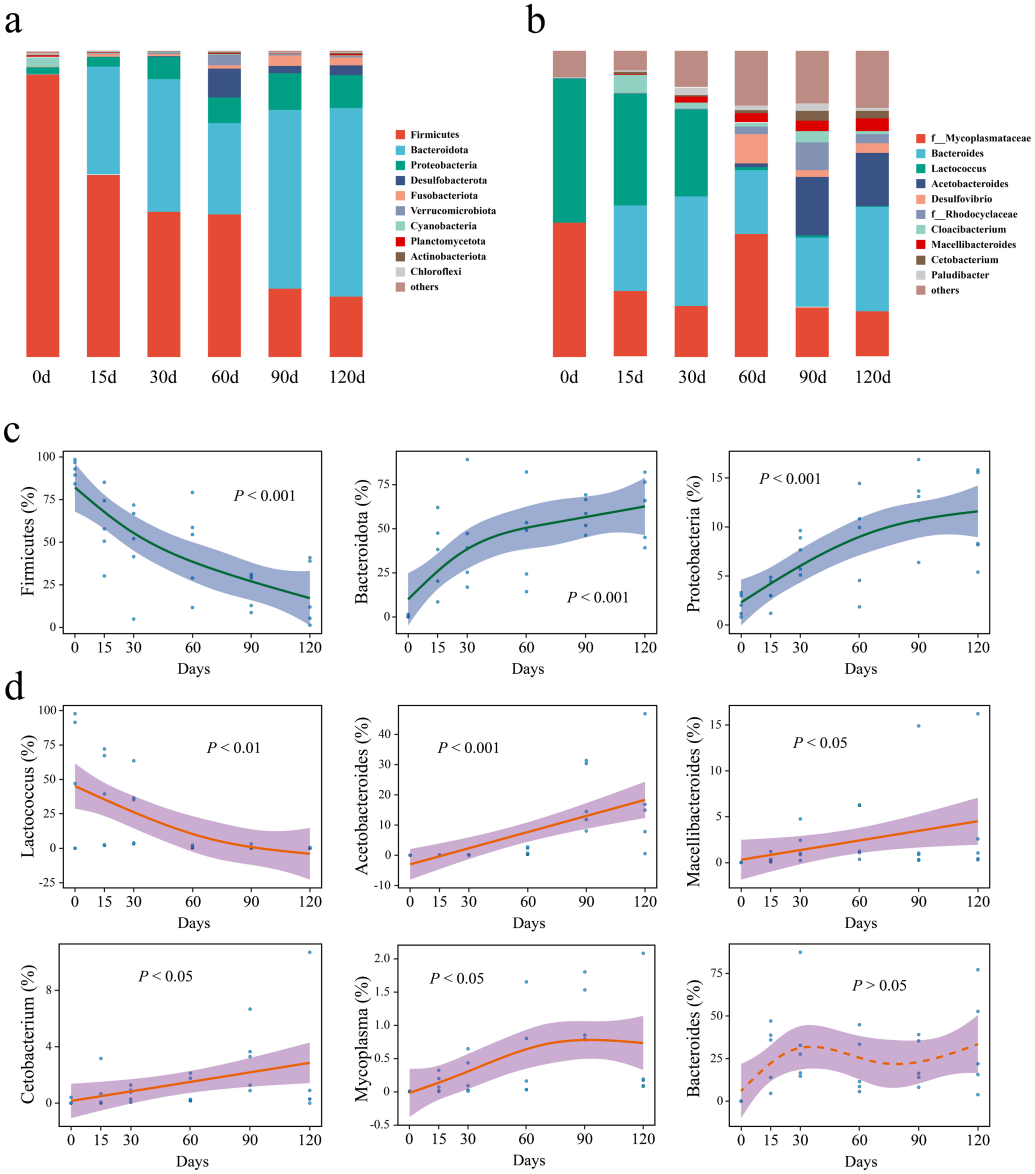
**(a,b)** Gut bacterial community composition of *P. canaliculata* snails during hibernation. **(c)** Key phyla of microorganisms in the snails gut during hibernation. **(d)** Key genera of microorganisms.

Before hibernation initiation (day 0), *Firmicutes* was the dominant phylum in the gut microbiota (Figure 3a). The dominant phylum gradually shifted from *Firmicutes* to *Bacteroidota* (*Bacteroidetes*) with increasing hibernation duration (Figure 3a). At the genus level, before hibernation, *Lactococcus* was the dominant genus (Figure 3b). The dominant genus gradually shifted from *Lactococcus* to *Bacteroides* with increasing hibernation duration (Figure 3b).

At the phylum level, the relative abundance of *Firmicutes* showed a significant relationship with hibernation duration (Figure 3c). Its relative abundance decreased from 92.3% before hibernation (day 0) to 19.7% after 120 days of hibernation. In contrast, the relative abundances of *Bacteroidota* and *Proteobacteria* significantly increased with the duration of hibernation (Figure 3c). The relative abundance of *Bacteroidota* and *Proteobacteria* increased from 0.4% and 2.1% before hibernation to 61.7% and 10.6% after 120 days of hibernation, respectively.

At the genus level, *Lactococcus* showed a pronounced decline in relative abundance with increasing hibernation duration (Figure 3d), dropping from 47.2% before hibernation to just 0.2% after 120 days. In contrast, the genera *Acetobacteroides*, *Macellibacteroides*, *Cetobacterium*, and *Mycoplasma* exhibited significant increases. Notably, *Acetobacteroides* increased from a negligible 0.0008% at day 0 to 17.4% after 120 days of hibernation.

**Figure 4:**
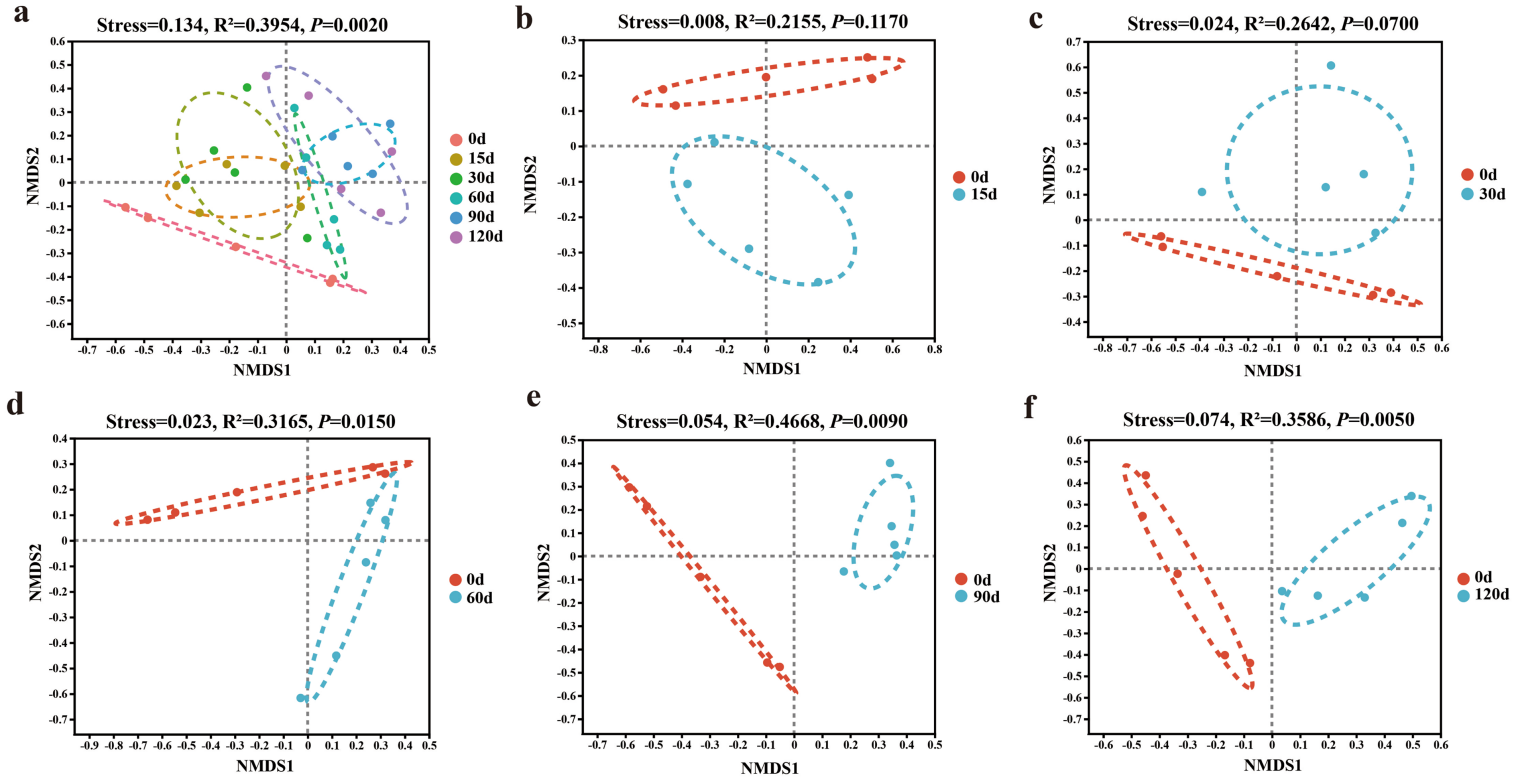
Gut microbial community structure **(a–f)** of *P. canaliculata* snails during hibernation. NMDS: Non-metric multidimensional scaling.

The NMDS plot and PERMANOVA analysis revealed that hibernation duration significantly influenced gut microbiota composition (Figure 4a). The intestinal bacterial community structure of snails at the 60th, 90th, and 120th days of hibernation showed significant differences compared with that before hibernation (Figures 4d–f). The intestinal bacterial community structure changed after 60 days of hibernation.

### 3.4 Differences in intestinal microbiome in across time points

**Figure 5:**
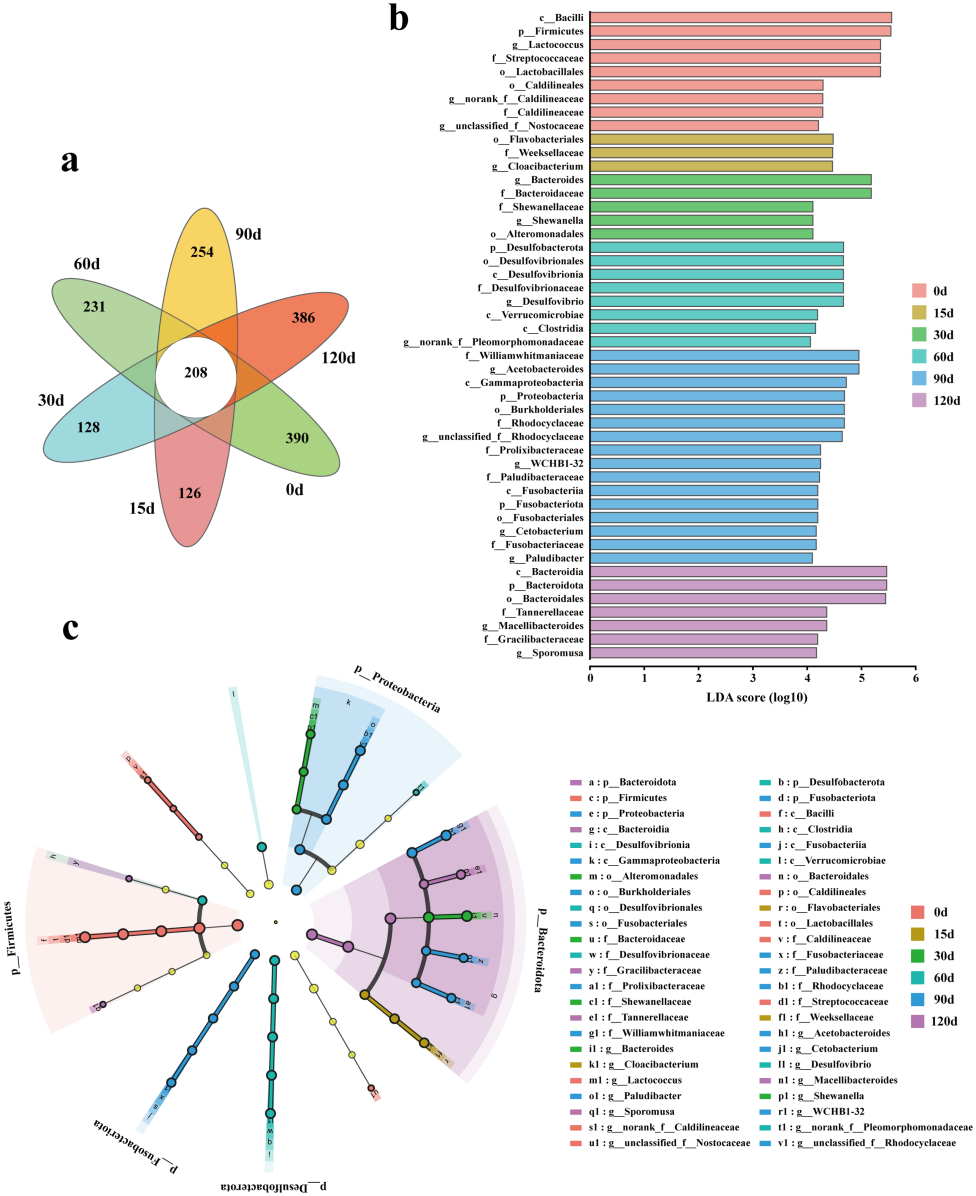
**(a)** Petal diagrams of OTU levels in gut microbes of *P. canaliculata* snails during hibernation. **(b,c)** LEfSe analysis of the snail gut microbes during hibernation (|LDA| > 4, *p* < 0.05). c, class; g, genus; f, family; o, order; p, phylum.

During the 120-day hibernation period, a total of 208 OTUs were shared across all six time periods (Figure 5a). Additionally, there were 390, 126, 128, 231, 254, and 386 unique OTUs specifically presenting in the snail gut microbiota at the 0th, 15th, 30th, 60th, 90th, and 120th days of hibernation, respectively (Figure 5a).

LEfSe was used to compare the impact of different hibernation periods on the gut microbiota (Figures 5b,c). The histogram of LDA scores revealed clear differences in microbial abundance among time points. The phylum *Firmicutes*, class *Bacilli*, order *Lactobacillales*, family *Streptococcaceae*, and genus *Lactococcus* were much enriched at the pre-hibernation period (Figures 5b,c). The order *Flavobacteriales*, family *Weeksellaceae*, and genus *Cloacibacterium* significantly enriched at 15th day of hibernation (Figures 5b,c). Similarly, the families *Bacteroidaceae* and *Shewanellaceae*, and genus *Bacteroides* enriched much at 30th day of hibernation (Figures 5b,c). The phylum *Desulfobacterota*, class *Desulfovibrionia*, order *Desulfovibrionales*, family *Desulfovibrionaceae*, and genus *Desulfovibrio* were significantly enriched at 60th day of hibernation (Figures 5b,c). The phylum *Proteobacteria*, class *Gammaproteobacteria*, order *Burkholderiales*, families *Williamwhitmaniaceae* and *Rhodocyclaceae*, and genus *Acetobacteroides* enriched much at 90th day of hibernation (Figures 5b,c). The phylum *Bacteroidota*, class *Bacteroidia*, order *Bacteroidales*, family *Tannerellaceae*, and genus *Macellibacteroides* significantly enriched at 120th day of hibernation (Figures 5b,c).

### 3.5 Changes in microbial community assembly during hibernation

**Figure 6:**
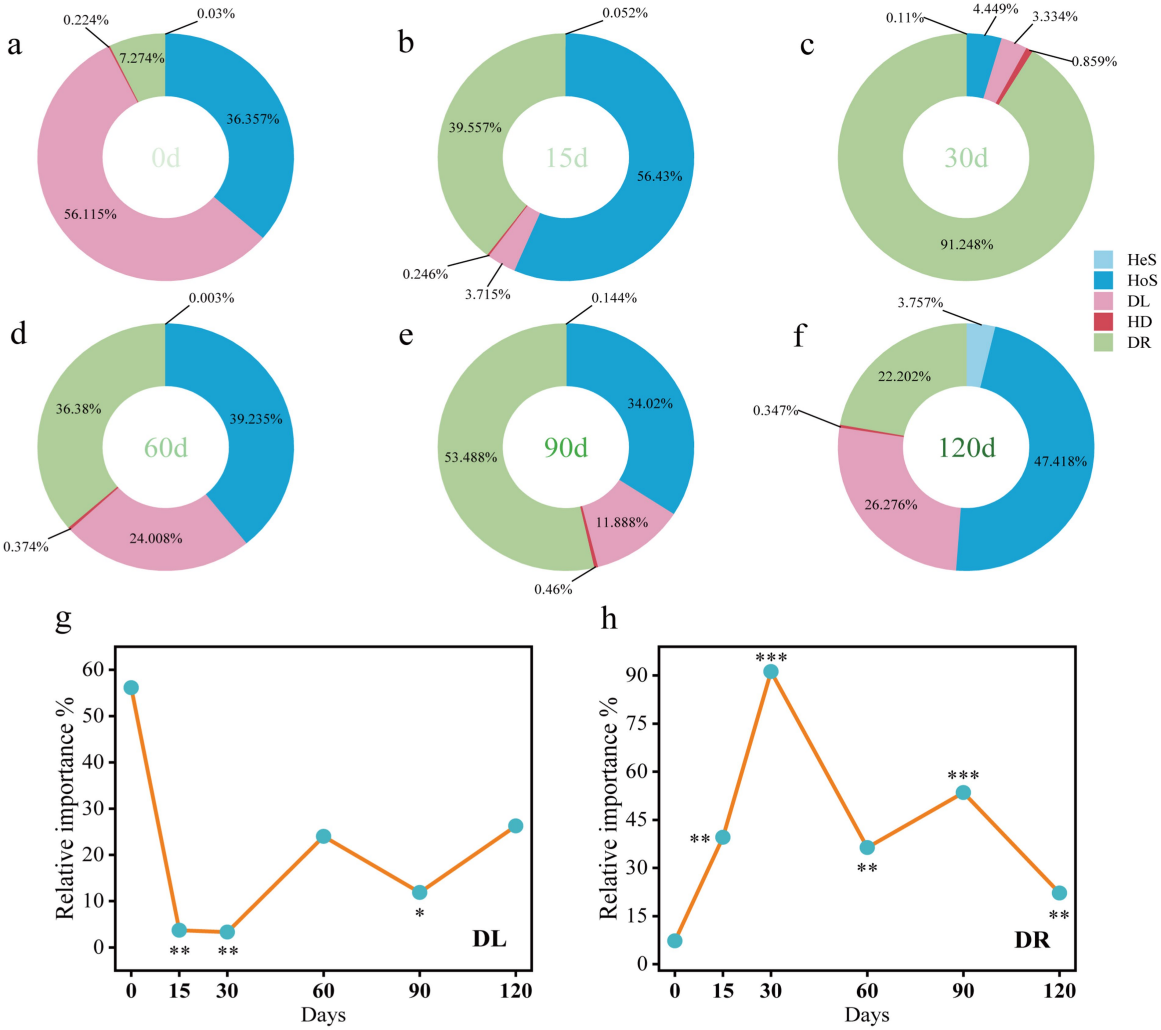
Ecological processes **(a–f)** of gut bacterial community assembly in *P. canaliculata* snails during hibernation. **(g)** Dispersal limitation. **(h)** Drift (and others). HoS, Homogeneous selection; HeS, Heterogeneous selection; HD, Homogenizing dispersal; DL, Dispersal limitation; DR, Drift (and others). *Represents significant difference in ecological process between post-hibernation and pre-hibernation. One-side significance based on bootstrapping test was expressed as *p < 0.1, **p < 0.05, ***p < 0.01.

The normalized stochasticity ratio (NST) was used to quantify the relative contributions of deterministic (niche-based) and stochastic (neutral) processes in shaping gut microbiota during hibernation (Supplementary Figure S2). Prior to hibernation, NST values averaged 32.5%, remaining below the 50% threshold, indicating that deterministic processes predominated in gut microbial community assembly. In contrast, during hibernation (15th to 90th days), NST values exceeded 50%, suggesting a shift toward stochastic dominance in community assembly. Gut bacterial community assembly in snails involved five ecological processes, with three being predominant: homogeneous selection, dispersal limitation, and drift (Figure 6). Notably, the contribution of drift significantly increased during hibernation (15th to 120th days) compared with pre-hibernation levels (Figure 6h). In contrast, dispersal limitation showed a significant decline at several time points during hibernation (15th, 30th, and 90th days) relative to pre-hibernation (Figure 6g).

### 3.6 Changes in phenotypic properties during hibernation

**Figure 7:**
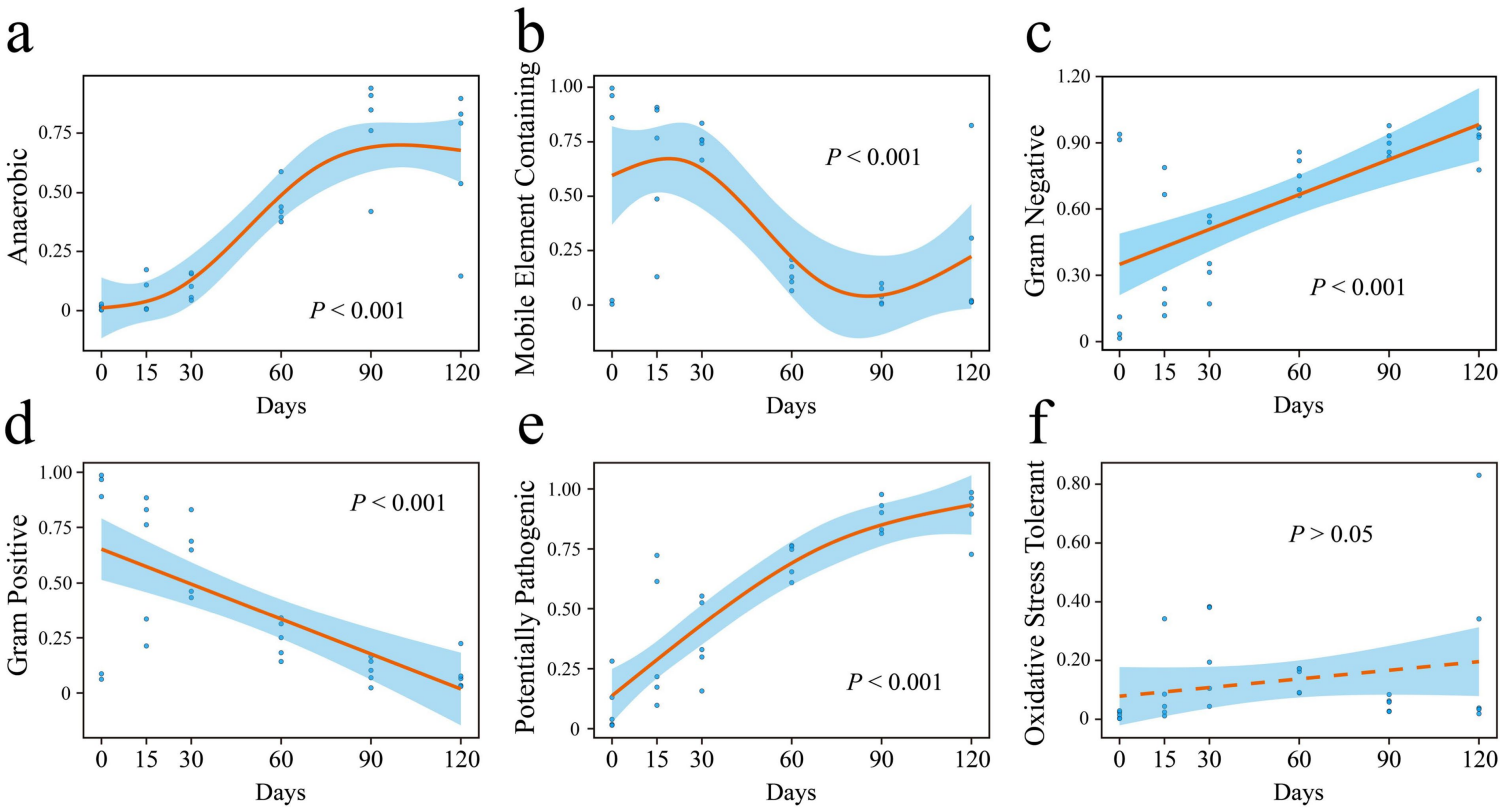
Phenotypic properties **(a–f)** of *P. canaliculata* gut microbes during hibernation predicted by BugBase. Y-axis represents the relative abundance of different phenotypic bacteria.

Based on phenotypic trait predictions from BugBase, the relative abundances of mobile element-containing and Gram-positive bacteria significantly declined with increasing hibernation duration (Figures 7b,d). In contrast, the relative abundances of anaerobic, Gram-negative, and potentially pathogenic bacteria significantly increased over time (Figures 7a,c,e). No significant correlation was observed between the relative abundance of oxidative stress-tolerant bacteria and hibernation duration (Figure 7f).

## 4 Discussion

### 4.1 Changes in alpha diversity of gut microbiota in *Pomacea canaliculata* during hibernation

**Figure 8:**
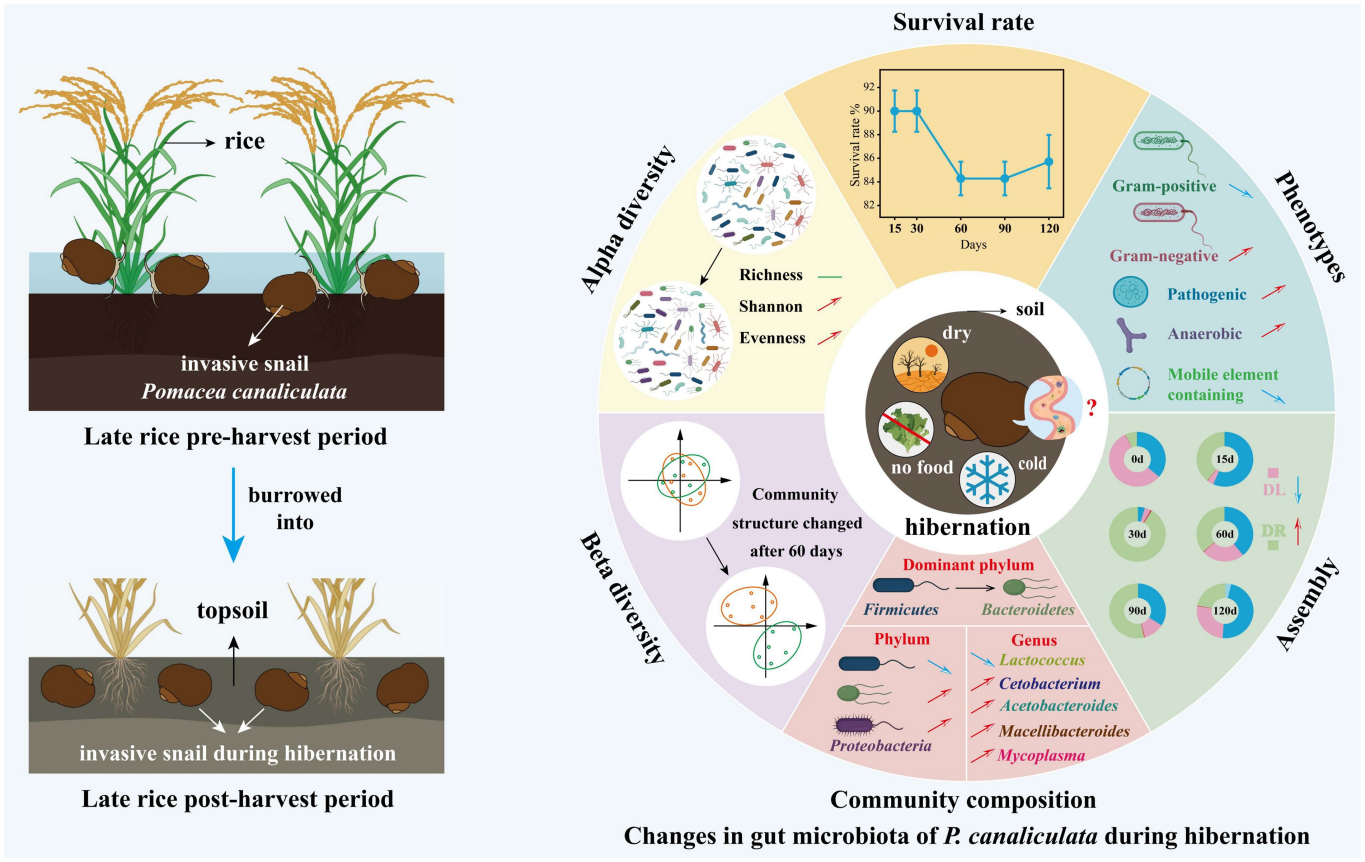
Schematic diagram revealing changes in gut microbiota of *P. canaliculata* snails during hibernation based on the obtained results. DL, Dispersal limitation; DR, Drift (and others). “↗” and “↘” represent positive and negative correlation with time, respectively. “↑” and “↓” represent increases and decreases, respectively.

Our findings indicated that the richness (Sobs and Chao1 indices) of gut microbiota remained unchanged after the snail entered hibernation (Figures 2a,d), while alpha diversity (Shannon index) increased with hibernation duration (Figure 2e). Moreover, the Pielou evenness of gut microbiota also increased over time (Figure 2c). These results suggest that during the hibernation of *P. canaliculata*, while the number of species in its gut microbiota remained relatively stable, the relative abundance distribution of species changed significantly with hibernation duration, leading to an increase in alpha diversity (Figure 8). Consequently, the observed increase in the Shannon index with hibernation duration may indicate an enhanced capacity of gut microbiota in *P. canaliculata* to adapt to external disturbances during hibernation (Stoffel et al., 2020). Zhou J. et al. (2022) revealed that gut microbiota alpha diversity (Shannon index) in the vertebrate Siberian chipmunk (*Tamias sibiricus*) was significantly higher after hibernation (from December to March) than pre-hibernation (November), which aligns with the results of this experiment. Similarly, Bosmans et al. (2018) also observed that gut microbiota alpha diversity (Shannon index) in the invertebrate *Bombus terrestris* was significantly higher after 16 weeks of artificial hibernation at 3°C than that of the non-hibernating group. Conversely, most previous studies supported that the alpha diversity (Shannon index) of gut microbiota in hibernating animals was typically lower during hibernation compared with active periods. Examples include mammals like *Urocitellus parryii* (Stevenson et al., 2014), *Ictidomys tridecemlineatus* (Carey et al., 2013; Dill-McFarland et al., 2014), bear *Ursus arctos* (Sommer et al., 2016), and *Rhinolophus ferrumequinum* (Xiao et al., 2019), as well as amphibians such as *Rana dybowskii* (Tong et al., 2019), *Polypedates megacephalus* (Weng et al., 2016), and *Strauchbufo raddei* (Cao et al., 2023). This study showed that during hibernation, the gut microbiota of snails exhibited an increase in alpha diversity due to a rise in evenness, which differed from the traditional pattern of decreased alpha diversity observed in hibernating animals. This observed difference could potentially stem from the remarkable hibernation adaptability and resilience exhibited by the invasive snail *P. canaliculata*.

### 4.2 Changes in beta diversity and composition of gut microbiota in *Pomacea canaliculata* during hibernation

Short-term hibernation (15–30 days) had a limited effect on the gut microbiota community structure of snails (Figure 4). However, long-term hibernation (60–120 days) significantly altered the community structure (Figure 8). Similarly, in some animals, the beta diversity of gut microbiota during hibernation significantly differed from pre-hibernation or non-hibernation periods, indicating pronounced alterations in gut microbiota structure during post-hibernation. For instance, in mammals, examples include *U. parryii* (Stevenson et al., 2014), *I. tridecemlineatus* (Dill-McFarland et al., 2014), *U. arctos* (Sommer et al., 2016), *R. ferrumequinum* (Xiao et al., 2019), and *Tamias sibiricus* (Zhou J. et al., 2022); in amphibians such as *R. dybowskii* (Tong et al., 2020) and *P. megacephalus* (Weng et al., 2016); and in invertebrates like *Bombus terrestris* (Bosmans et al., 2018).

Before hibernation, *Firmicutes* dominated the snail gut microbiota, accounting for 92.3% of the relative abundance (Figure 3a). During the 15–60 days of hibernation, *Bacteroidota* also became dominant, reaching approximately equal relative abundance to that of *Firmicutes* (Figure 3a). As hibernation progressed to 90–120 days, the dominant phylum shifted from *Firmicutes* to *Bacteroidota*, with the latter reaching a relative abundance of approximately 60% (Figures 3a, 8). Previous studies have consistently found that *Bacteroidota*, *Firmicutes*, and *Proteobacteria* are among the three most dominant phyla (not in any particular order) in the gut microbiota of hibernating animals during their hibernation period (Sonoyama et al., 2009; Wiebler et al., 2018; Tong et al., 2020). Notably, *Bacteroidota* often emerges as the most dominant phylum during hibernation (Dill-McFarland et al., 2014; Sommer et al., 2016; Weng et al., 2016; Tong et al., 2019).

### 4.3 Key gut microbes of *Pomacea canaliculata* during hibernation

The relative abundance of phylum *Firmicutes* in gut microbiota of snails significantly decreased with hibernation duration (Figures 3c, 8). In contrast, phyla *Bacteroidota* and *Proteobacteria* relative abundances significantly increased with hibernation duration (Figures 3c, 8). A large body of prior research supports the trend of decreased relative abundance of *Firmicutes* in snail gut microbiota during hibernation compared with that at pre-hibernation or non-hibernation periods, consistent with the findings of this study. Examples include *U. parryii* (Stevenson et al., 2014), *I. tridecemlineatus* (Dill-McFarland et al., 2014), *P. megacephalus* (Weng et al., 2016), *R. dybowskii* (Tong et al., 2019), among others (Sommer et al., 2016; Xiao et al., 2019). Moreover, these studies also observed a general increase in the relative abundances of *Bacteroidota* and *Proteobacteria* during hibernation. The rising relative abundance of the phylum *Bacteroidota* during hibernation might be attributed to the ability of *Bacteroidota*-related bacteria to metabolize host polysaccharides, enabling them to thrive in the absence of food in the gut during hibernation (Sonnenburg et al., 2005). Additionally, *Bacteroidota*-related bacteria may also participate in metabolizing proteins and fats provided by the gut epithelium (Wu et al., 2011). The decline in the relative abundance of *Firmicutes* might be linked to the lack of relevant food sources during hibernation. As food was digested, the proportion of bacteria from the *Firmicutes* phylum involved in the metabolism of plant polysaccharides (Boutard et al., 2014) and carbohydrates (Garbacz, 2022), such as *Clostridia* class and *Lactobacillales* order, may decrease.

The relative abundance of genus *Lactococcus* decreased significantly with hibernation duration (Figures 3d, 8). Before hibernation, its relative abundance was 47.2%, but after 120 days of hibernation, it dropped to less than 1%. This observation aligns with the significant decrease in *Streptococcaceae* during hibernation in some animals, as *Lactococcus* belongs to this family (Sommer et al., 2016; Tong et al., 2019). *Lactococcus* is primarily involved in carbohydrate metabolism, and the fasting state during hibernation may lead to a dramatic decrease in the proportion of this genus (Buron-Moles et al., 2019). The decrease in the relative abundance of *Firmicutes* after hibernation may be related to the sharp decline in the relative abundance of *Lactococcus* within this phylum. In contrast, the relative abundance of family *Lachnospiraceae* increased slightly over the course of hibernation. This finding is contrary to some previous studies (Dill-McFarland et al., 2014; Stevenson et al., 2014), which reported decreases in *Lachnospiraceae* during hibernation.

SCFAs are major metabolites of the intestinal microbiota, which not only provide energy for intestinal epithelial cells, but also stimulate the proliferation of intestinal mucosal cells to maintain gut health (Peng et al., 2009; Donohoe et al., 2011; Fukuda et al., 2012; Morrison and Preston, 2016). The relative abundances of genera *Acetobacteroides*, *Macellibacteroides*, and *Cetobacterium* exhibited a significant increase during snail hibernation (Figure 3d). These organisms play a crucial role in metabolizing host polysaccharides to produce SCFAs such as acetate and propionate (Tsuchiya et al., 2008; Jabari et al., 2012; Zhang et al., 2015; Liu et al., 2023). These SCFAs serve as a vital energy source for the host during hibernation while also stimulating the secretion of intestinal mucin. Similarly, the relative abundances of the genus *Bacteroides* substantially rose during the hibernation period from 15th to 120th day (Figure 3a). Like the aforementioned genera, *Bacteroides* also actively participates in the metabolism of host polysaccharides to generate SCFAs (Sonnenburg et al., 2005; Rios-Covian et al., 2017). The collective surge in the abundance of these four genera, all belonging to the phylum *Bacteroidota*, stands as the primary driver behind the notable increase in the relative abundance of this phylum throughout the hibernation period. In addition, the relative abundance of genus *Mycoplasma* increased slightly during hibernation (Figures 3d, 8), which may be related to the decreased immune function of the snails in the dormant state.

### 4.4 Changes in assembly and phenotypes of gut microbiota in *Pomacea canaliculata* during hibernation

Deterministic and stochastic processes are thought to play simultaneous roles in the assembly of microbial communities (Chase, 2010; Ning et al., 2020). In this study, deterministic processes dominated the assembly of the gut microbiota in snails before hibernation (Supplementary Figure S2), a pattern consistent with observations in shrimp (Xiong et al., 2017). In contrast, for sea cucumbers, *Drosophila simulans* (fly), and *Dicranocephalus wallichii bowringi* (beetle), gut microbiota assembly was mainly controlled by stochastic processes (Zhao et al., 2022; Zhu et al., 2022). These findings reflect that the community assembly of gut microbiota may be influenced by host species. However, some recent studies indicated that microbial community assembly is also related to geographic location, pH, temperature, and other environmental factors (Martinson et al., 2017; Jiao and Lu, 2019). Our results demonstrated that hibernation altered the assembly of the snail gut microbiota (Figure 6; Supplementary Figure S2). During hibernation, stochastic processes (e.g., ecological drift) dominated the assembly of gut microbiota, resulting in divergent temporal succession of microbial communities. In this study, the relative importance of dispersal limitation in the gut microbiota of snails during hibernation decreased, indicating that the restrictions on the transmission or migration of gut microbes within the snails reduced (Zhou and Ning, 2017). The increased diversity of the snail gut microbiota during hibernation may be associated with the decline in dispersal limitation.

We found that the abundance of anaerobic bacteria in the gut of hibernating snails increased significantly with hibernation duration (Figures 7a, 8). During hibernation, the slowed metabolism and reduced gut motility of snails resulted in an anaerobic environment in the gut, leading to a substantial increase of anaerobic bacteria. Some anaerobes can ferment to produce acids, providing energy for the hibernating host and protecting the intestinal mucosa (Sonoyama et al., 2009; Sommer et al., 2016). Interestingly, before hibernation, the snail gut enriched with abundant mobile elements (Figure 7b). However, studies have shown that these mobile elements are significantly positively correlated with intrinsic antibiotic resistance genes (Su et al., 2020). A gut metagenomic study of *P. canaliculata* showed that its gut microbiota had resistance against environmental pollution stresses like heavy metals and pesticides (Liu et al., 2018). This is analogous to the stress resistance of snails before hibernation in this experiment. But after hibernation, bacteria carrying mobile elements gradually decreased over time. Notably, the potential pathogenicity of the gut microbiota significantly increased with the duration of hibernation (Figure 7e). During hibernation, the metabolic rate of snails decreases to conserve energy, which may compromise their immune system (e.g., through reduced hemolymph circulation and suppressed immune cell activity), thereby diminishing pathogen defense. In addition, the reduced dispersal limitation in the assembly of the gut microbiota may facilitate the invasion and enrichment of pathogens. However, in this study, even after prolonged hibernation (up to 120 days), the snails exhibited a high survival rate of 85%. This suggests that the invasive snail *P. canaliculata* exhibits unique adaptive strategies during hibernation, enabling it to tolerate the enrichment of intestinal pathogens.

Overall, after the invasive snail *P. canaliculata* enters hibernation, significant changes occur in the diversity, composition, phenotypic characteristics, and community assembly of its gut microbiota. Post-hibernation, the snails are in a fasting state, and nutrients such as carbohydrates in the intestinal contents are gradually depleted, leading to substantial alterations in the gut environment. This may drive adaptive changes in the gut microbiota to cope with the harsh conditions during hibernation.

## 5 Conclusion

In this study, we investigated the dynamics of gut microbiota in the invasive snail *P. canaliculata* during a 120-day hibernation period. Notably, 85.7% of the individuals survived the entire duration, indicating strong hibernation tolerance. Our results demonstrated that both alpha diversity (Shannon index) and community evenness (Pielou index) of the gut microbiota increased over time. Beta diversity analysis revealed a significant shift in the gut microbial community structure after 60 days of hibernation compared with that at pre-hibernation. The dominant phylum shifted from *Firmicutes* to *Bacteroidota* with increasing hibernation duration, with *Bacteroides* emerging as the predominant genus. The relative abundances of *Firmicutes* and genus *Lactococcus* declined markedly, while those of phyla *Bacteroidota* and *Proteobacteria*, and genera *Acetobacteroides*, *Macellibacteroides*, *Cetobacterium*, and *Mycoplasma* increased throughout the hibernation period. Changes in the gut bacterial community were closely associated with stochastic assembly processes. Phenotypic trait predictions further revealed an increase in anaerobic and potentially pathogenic bacteria, accompanied by a decline in mobile genetic elements within the gut microbiome during hibernation. Collectively, these findings suggest that *P. canaliculata* undergoes significant gut microbiota remodeling during hibernation, which may contribute to its metabolic resilience and invasive success.

## Data Availability

The original contributions presented in the study are publicly available. This data can be found here: https://www.ncbi.nlm.nih.gov, accession number PRJNA1152916.
