# Supplementary material for: Dynamics in gut microbiota diversity, composition, and assembly reveal the adaptability of invasive snail Pomacea canaliculata during hibernation in rice fields
Source: Front Microbiol. 2025 Jul 16;16:1616681. doi: 10.3389/fmicb.2025.1616681 (PMC12307439; doi:10.3389/fmicb.2025.1616681)
Supplement: Supplementary file 1 [file Supplementary_file_1.docx]

Supplementary Material


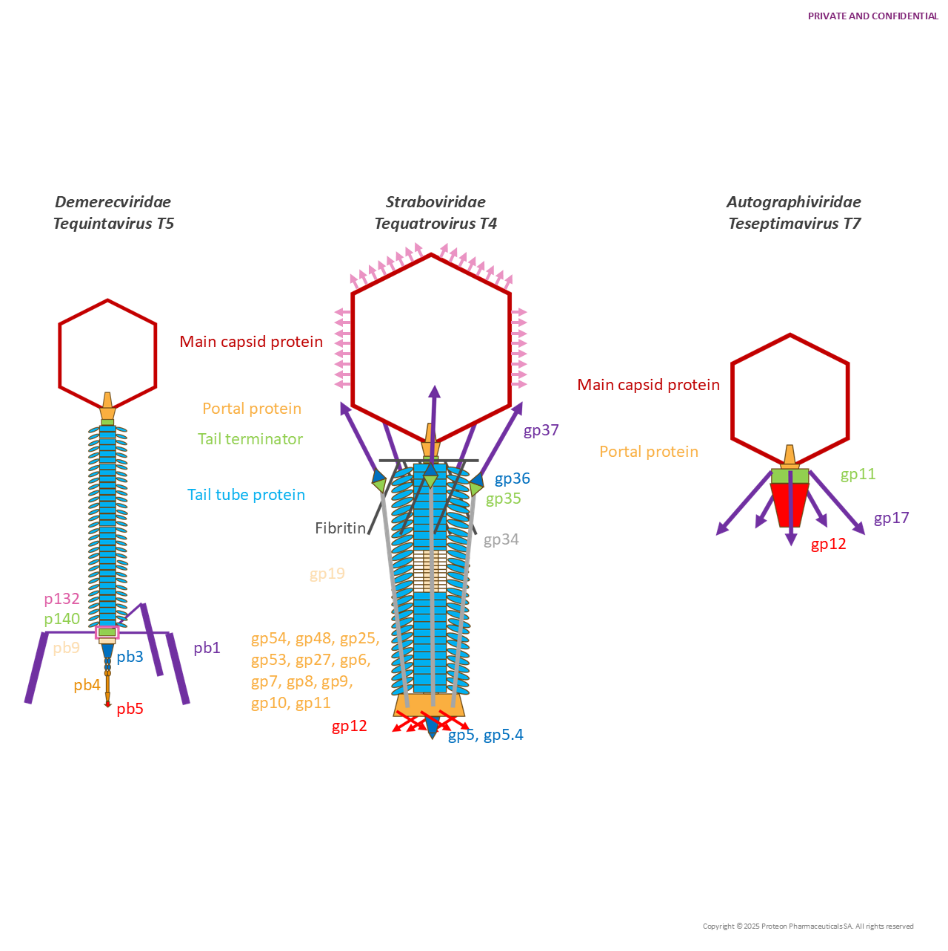


Supplementary Figure 1. Comparison of architecture examples of bacteriophages belonging to three different families of the class *Caudoviricetes*.


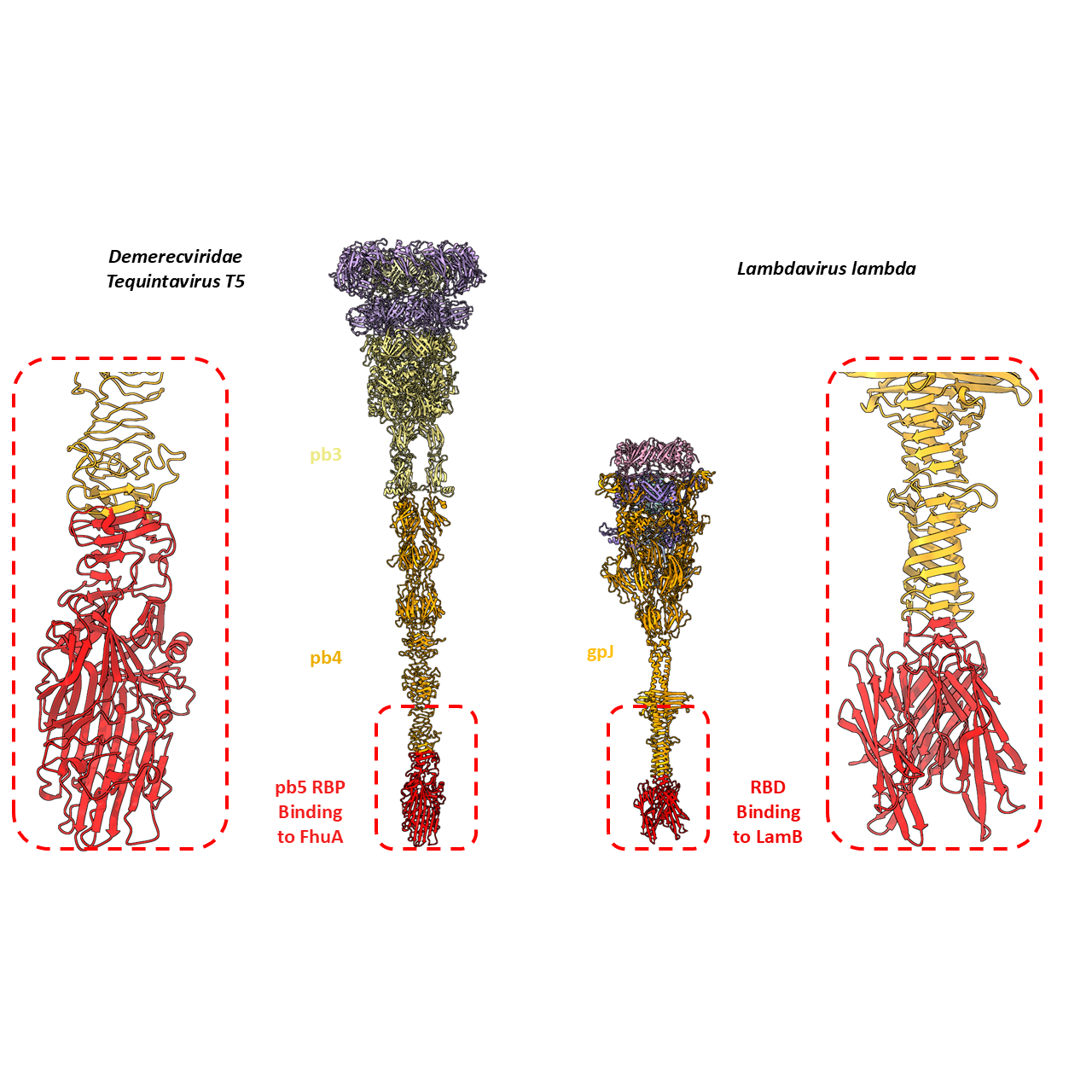


Supplementary Figure 2. The comparison of reconstructions of *Tequintavirus T5* and *Lambdavirus* *lambda* receptor binding proteins of tail spikes. The receptor binding proteins or receptor binding domains are presented in red, a flexible tail spikes which change structure after binding to receptors are in orange and yellow. The digital reconstruction of *Tequintavirus T5* tail spike is based on the PDB structure 7zqb. The interaction between pb4 and pb5 was reconstructed manually based on homology modelling and protein complex modelling by Alphafold2-Multimer in this study. The Unresolved domains in the PDB structure (8k35) of the *Lambdavirus lambda* tail spike was reconstructed also by Alphafold2-Multimer.


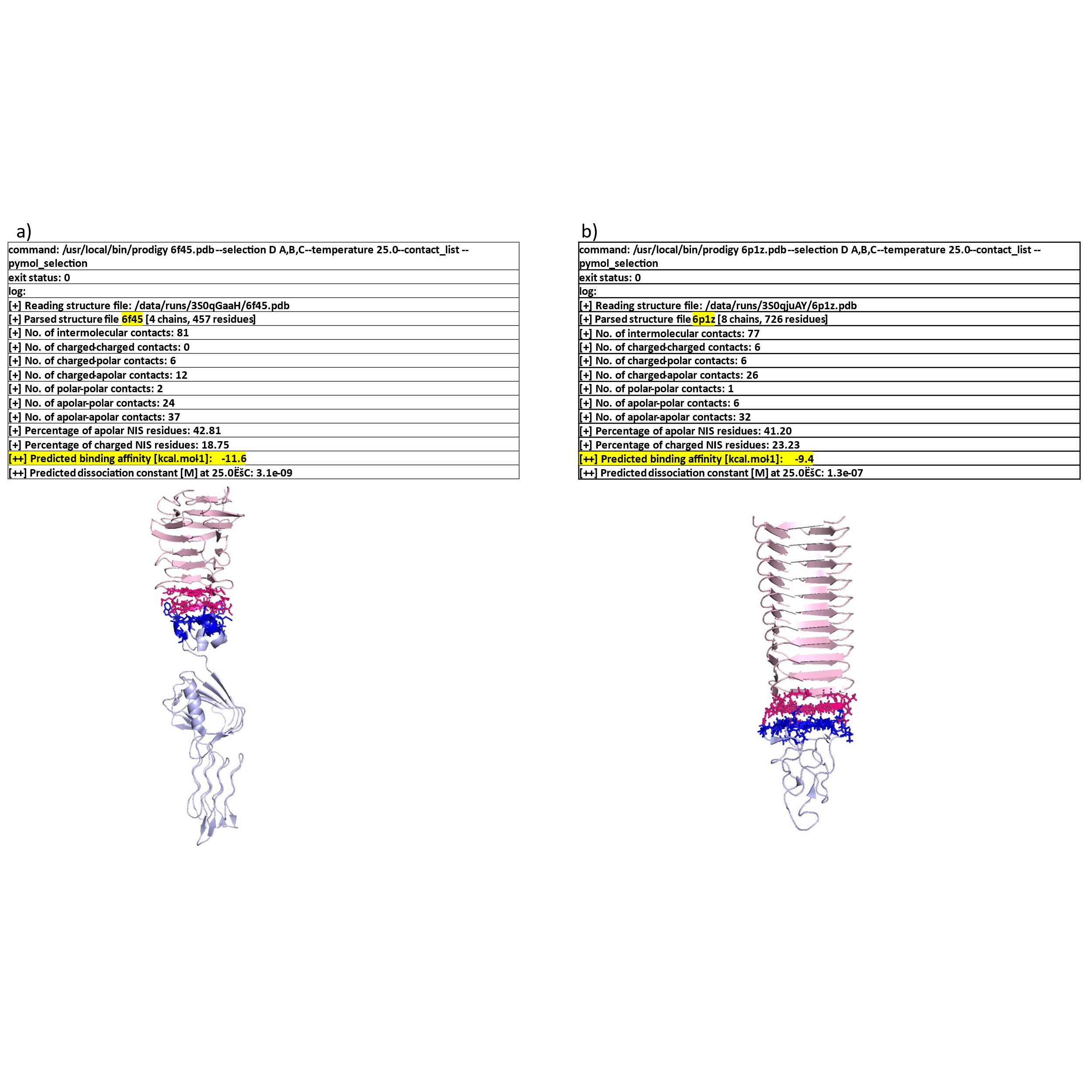


**Supplementary Figure 3.** The estimation of binding affinity natural existing interaction between receptor binding protein and the tail fibre (A) and tail tip protein gp5.4 from T4 phage and the tail spike lysozyme (B).


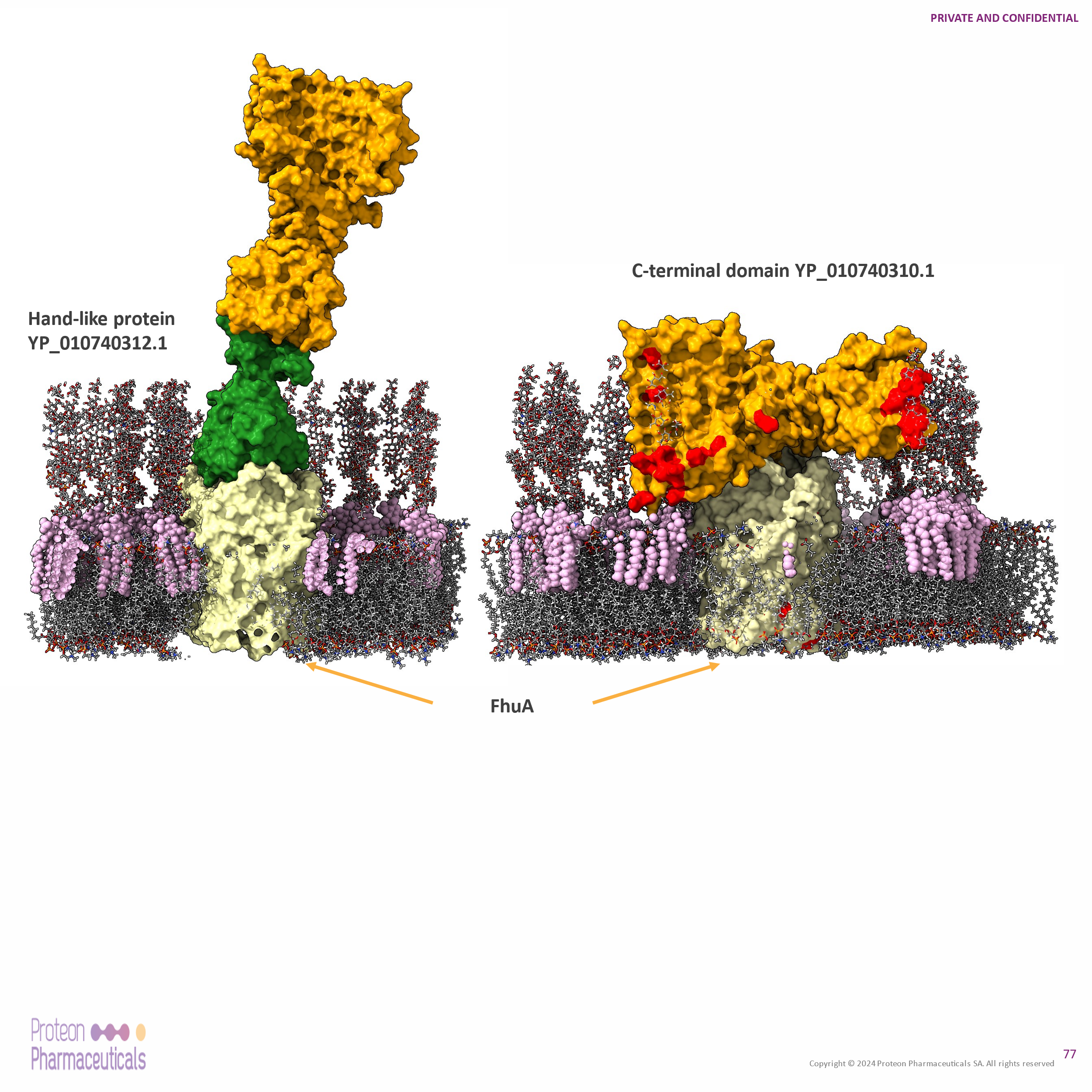


**Supplementary Figure 4.** The digital reconstruction of *E. coli* cell membrane with FhuA proteins and *Dhillonvirus JLBYU60*’s tail spike ended with the Hand-like protein or without. The red spot represents clashes with LPS sugars*.*


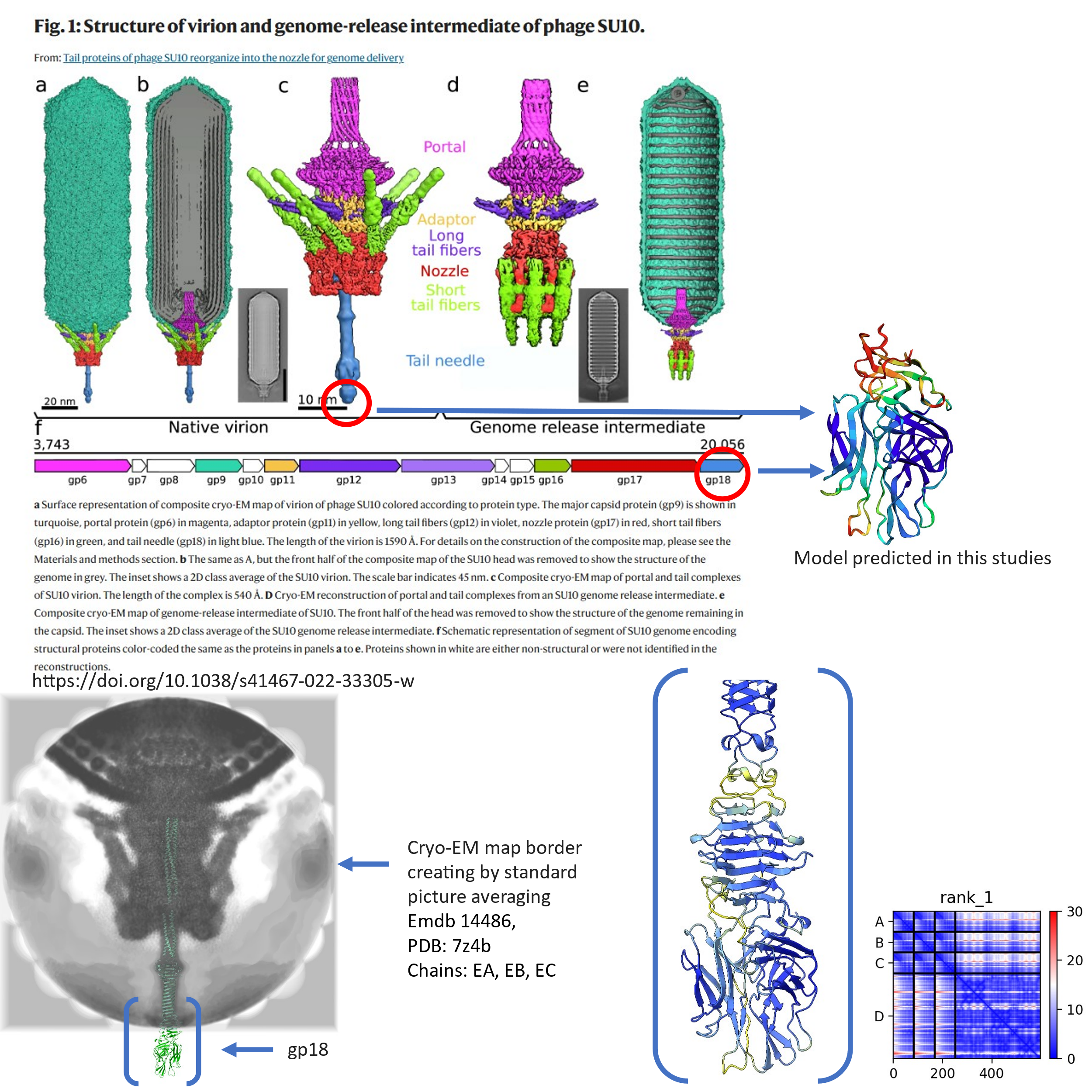


**Supplementary Figure 5.** *Kuravirus SU10* Cryo-EM map 14486 with fitted PDB structure 7z4b chains: EA, EB, EC, our hypothesis suggest that hand-like protein (gp18) is beyond collected data collected during Cryo-EM map processing. The AF2 model of SU10 tail spike end with attachment hand-like protein and PAE error matrix*.*
